# Supplementary material for: Socioeconomic characteristics, family structure and trajectories of children’s psychosocial problems in a period of social transition
Source: PLoS One. 2020 Jun 5;15(6):e0234074. doi: 10.1371/journal.pone.0234074 (PMC7274440; doi:10.1371/journal.pone.0234074)
Supplement: Attachment — Submitted filename: Response to Reviewers.docx [file pone.0234074.s002.docx]

**Dear Sir or Madam,**

**We would like to thank you for your feedback. We appreciate the detailed response and suggestions for improving our paper. We attempted to incorporate most of the comments and believe that the manuscript quality improved. To ensure proper use of English, the text was revised by a professional editor.**

**Please find our answers to the individual comments as blue bulleted points below.**

**Reviewers' comments:**

Reviewer's Responses to Questions

Comments to the Author

1. If the authors have adequately addressed your comments raised in a previous round of review and you feel that this manuscript is now acceptable for publication, you may indicate that here to bypass the “Comments to the Author” section, enter your conflict of interest statement in the “Confidential to Editor” section, and submit your "Accept" recommendation.

Reviewer #1: (No Response)

Reviewer #2: All comments have been addressed

2. Is the manuscript technically sound, and do the data support the conclusions?

The manuscript must describe a technically sound piece of scientific research with data that supports the conclusions. Experiments must have been conducted rigorously, with appropriate controls, replication, and sample sizes. The conclusions must be drawn appropriately based on the data presented.

Reviewer #1: Yes

Reviewer #2: Yes

3. Has the statistical analysis been performed appropriately and rigorously?

Reviewer #1: Yes

Reviewer #2: Yes

4. Have the authors made all data underlying the findings in their manuscript fully available?

The PLOS Data policy requires authors to make all data underlying the findings described in their manuscript fully available without restriction, with rare exception (please refer to the Data Availability Statement in the manuscript PDF file). The data should be provided as part of the manuscript or its supporting information, or deposited to a public repository. For example, in addition to summary statistics, the data points behind means, medians and variance measures should be available. If there are restrictions on publicly sharing data—e.g. participant privacy or use of data from a third party—those must be specified.

Reviewer #1: Yes

Reviewer #2: (No Response)

5. Is the manuscript presented in an intelligible fashion and written in standard English?

PLOS ONE does not copyedit accepted manuscripts, so the language in submitted articles must be clear, correct, and unambiguous. Any typographical or grammatical errors should be corrected at revision, so please note any specific errors here.

Reviewer #1: Yes

Reviewer #2: Yes

6. Review Comments to the Author

Please use the space provided to explain your answers to the questions above. You may also include additional comments for the author, including concerns about dual publication, research ethics, or publication ethics. (Please upload your review as an attachment if it exceeds 20,000 characters)

Reviewer #1: The authors have undertaken substantial revision and done an excellent job in responding to the reviewer’s comments and as such, the manuscript is greatly improved. I have some further minor suggestions as below. The manuscript needs a second thorough review for grammatical errors – I have flagged some of these.

Abstract

Grammatical errors.

“Findings from western setting(s)…” and throughout the manuscript.

“Modelled as (a) quadratic growth curve”

“Essential for (a) more complex view”

Introduction

Line 42. “a decrease in (a) child’s psychosocial problems” (and Line 53)

Consider some rewording in the following places:

Line 48-49. Not all re-partnered families are married couples. There are plenty of studies that separate re-partnered from nuclear families – the focus on single parent families in family structure has shifted and multiple categories (including step and blended families) are standard in statistical collections.

- We agree that multiple studies provide a more detailed distinction between various family types. However, existing studies with a focus similar to ours have also chosen to use single-parent families as a single category, e.g. [5,6]. The section was re-written to reflect this more clearly.

Line 55. Explain the practical interpretation of the Flouri study for those not familiar with quadratic growth curves i.e. it’s not immediately obvious what a vertical shift of the growth curve means in respect to family structure and SES.

- Explanation added.

Line 69. Did the Kidscreen study [13] include family structure and or SES which would make this a more appropriate point to make – the effect of something like parental mental health is universal? You have noted lower SES in Eastern European countries but how did the mental health of Czech children, and the relationship of MH with SES compare with other countries?

- This information was added to the article. The entire paragraph was rephrased do reflect this more clearly.

Line 87. Why do you expect a smaller effect size? Suggest adding a because.. statement to the end of this sentence.

- Rationale added.

Materials and methods

Looks good.

The analytical sample varies across Models with the addition of family structure and education variables. Although the analytical sample is defined by SDQ score, please clarify if there are cases in the 3,261 that have no data on predictive variables at any follow up? That is, if I look at Table 2, the Ns are relevant for Model 1 but what about Models 2 & 3 when family structure and maternal education are added?

- In general, this is a valid concern. However, for the purposes of our study the mean SDQ scores remain stable even when the sample is reduced because of missing covariates. As a result, we decided not to include them. Another option would be to remove subjects with missing covariates from the analysis altogether. We decided against thus because such a choice does not affect the results in any way – except for the loss of statistical power.

Results

Line 196. Correction – “Unfortunately, from this point on the information on the non-analytic sample is limited”.

Table 1. It doesn’t make much sense to have data about family structure for the non-analytic sample beyond that measured at birth. Suggest removing these figures from the table.

- Redundant figures were removed from the table.

In terms of the analytical sample, I wonder if reporting the % of those in different family structures of the non- missing cases would be more informative i.e. keep the N for Missing but exclude from the denominator. Then we can easily see the change in family structure based on available cases albeit with likely biased attrition in the sample.

- The information on the proportion of the family structure types in non-missing cases only is presented in the %nmiss column.

You could also add the year to the row labels e.g. “Family structure at 7y (1998/99) to connect back to the period in time which is interesting given the dramatic change.

- Years corresponding to data points were added to the table.

In terms of assessing differential attrition in family types, this could be assessed based on drop outs from year 7 e.g. proportion of original, single and re-partnered families still in the sample at year 11 etc.. Because you have already excluded those with no SDQ data, this may not show a lot more.

- We considered this, but the difference is quite small. The majority of subjects had complete data until a specific time-point and then dropped out, which is mostly reflected in Table 1.

Discussion

Line 258. Are you able to report here or perhaps in the intro the increase in the proportion of single parent and re-partnered families during this time based on other sources?

- We decided not to incorporate this suggestion, because we believe that this information does not raise an interesting point and requires substantial change in the text.

Line 259. Be careful in this paragraph not to make it seem that all single parent families are burdened. Many children in single parent families also do well. (delete The) Single parenthood comes with.. “

- The sentence was modified.

Line 267. Reference [8] – was it the case in this study that internalizing problems were not connected to quality of relationship with the father?

- Yes, this study only found a link between externalizing problems and the quality of the relationship with the father. The association for the internalizing problems was small and not significant.

Line 270 “For (the) hyperactivity subscale..” Please explain this sentence in practical terms.. “mostly provided significant negative vertical shift, meaning that…” hyperactivity problems were lower in children of university educated mothers across ages”?

- The sentence was expanded to include the explanation.

Line 277. “higher socioeconomic status is a protective factor for prosocial behaviour” remove ‘the’

Line 283 “lower income is usually associated also with lower education” remove ‘the’

Line 292. “Provides (a) more comprehensive picture”

Line 296 Is it worth mentioning family structure and prosocial behaviour over time (less improvement in single parent and new partner families based on significant difference in curve?

- Initially, we did not include these results as they were borderline significant. However, they are quite visible in Fig 1, which was added with the revision and, therefore, is worth mentioning.

Line 303. “Comes from (a) longitudinal study..”

Line 313. Does your model meet assumptions? Mentioning this here is a concern unless you can report that it does meet assumptions in the Methods.

- Clarification added. The violation of these assumptions results in biased estimates for individual subjects but the fixed effects estimates remain unbiased.

Line 320. Further research – perhaps consider the total SDQ scale and internalising/externalising scales which tend to be more reliable than individual sub-scales in population samples (e.g. see Goodman et al., 2010).

- Thank you for pointing out this article, it does make more sense to use the internalising and externalising subscales when they have better properties in population samples. The suggestion was incorporated into the further research paragraph.

Reviewer #2: (No Response)
